# Research on factor analysis and method for evaluating grouting effects using machine learning

**DOI:** 10.1038/s41598-024-57837-x

**Published:** 2024-04-02

**Authors:** Wenxin Li, Juntao Chen, Jun Zhu, Xinbo Ji, Ziqun Fu

**Affiliations:** 1 Shandong Univ Sci & Technol, Coll Energy & Min Engn, Qingdao, People’s Republic of China; 2 Shandong Energy Xinwen Mining Group Suncun Coal Mine, Taian, 271219 China

**Keywords:** Grouting effect evaluation, Correlation analysis, Optimization algorithm, Machine learning, Hydrology, Engineering

## Abstract

The evaluation of grouting effects constitutes a critical aspect of grouting engineering. With the maturity of the grouting project, the workload and empirical characteristics of grouting effect evaluation are gradually revealed. In the context of the Qiuji coal mine’s directional drilling and grouting to limestone aquifer reformation, this study thoroughly analyzes the influencing factors of grouting effects from geological and engineering perspectives, comparing these with various engineering indices associated with drilling and grouting. This led to the establishment of a “dual-process, multi-parameter, and multi-factor” system, employing correlation analysis to validate the selected indices’ reasonableness and scientific merit. Utilizing the chosen indices, eight high-performing machine learning models and three parameter optimization algorithms were employed to develop a model for assessing the effectiveness of directional grouting in limestone aquifers. The model’s efficacy was evaluated based on accuracy, recall, precision, and F-score metrics, followed by practical engineering validation. Results indicate that the “dual-process, multi-parameter, multi-factor” system elucidates the relationship between influencing factors and engineering parameters, demonstrating the intricacy of evaluating grouting effects. Analysis revealed that the correlation among the eight selected indicators—including the proportion of boreholes in the target rock strata, drilling length, leakage, water level, pressure of grouting, mass of slurry injected, permeability properties of limestone aquifers before being grouted, permeability properties of limestone aquifers after being grouted—is not substantial, underscoring their viability as independent indicators for grouting effect evaluation. Comparative analysis showed that the Adaboost machine learning model, optimized via a genetic algorithm, demonstrated superior performance and more accurate evaluation results. Engineering validation confirmed that this model provides a more precise and realistic assessment of grouting effects compared to traditional methods.

## Introduction

The geological conditions of China’s coal mines are notably complex, making it one of the countries most afflicted by mine water damage globally. Aquifer modification through grouting represents a commonly utilized technique in the realm of mine water damage prevention and control, and evaluating the effects of such grouting is an indispensable and crucial component of these projects.

The broad application of grouting technology in mining has increasingly drawn the attention of scholars and engineers to the detection and evaluation of its effects. Currently, the most direct and effective methods for detecting and evaluating grouting effects include inspection hole, compression test, and direct pumping test methods in mines^1^. Xue et al.^2^ employed a combination of P-Q-t control, physical exploration, inspection hole, and digital drilling camera methods to thoroughly assess the grouting effects on the F4-4 gushing fault in Qingdao Jiaozhou Bay undersea tunnel. Ren et al.^3^ applied permeability detection methods to effectively evaluate karst foundation grouting effects, recommending 47 Lu (a permeability unit defined as 1 L of water injected per minute per meter of test section at 1 MPa water pressure) as the critical permeability threshold for potential erosion and collapse. Liu et al.^4^ achieved visualization of residual water influx data post-grouting via mapping, thereby optimizing evaluation criteria and visually representing grouting effects. Zhang et al.^5^ analyzed temporal changes in grouting pressure and volume to assess grouting effects. These studies predominantly focus on utilizing one or several of the aforementioned testing methods for grouting effect evaluation. They often involve single-factor assessments, are labor-intensive and costly, and typically yield only preliminary qualitative analyses, thereby making it challenging to obtain accurate and comprehensive evaluation results.

To address these issues, experts and scholars have developed more systematic and comprehensive evaluation methods, integrating various comprehensive assessment techniques: Sima^6^ applied fuzzy comprehensive evaluation theory, coupled with the Analytic Hierarchy Process (AHP), to ascertain parameter weights and establish a mathematical model for grouting effect evaluation, successfully applying it to the curtain grouting project in Carp Naihu mine. Hou et al.^7^ proposed a fuzzy comprehensive evaluation method based on gray correlation degree, reversed the use of gray correlation degree method to construct the initial matrix, and combined with the principle of AHP and fuzzy comprehensive evaluation, and came to the conclusion that the grouting effect of Guiyang subway tunnel grouting and plugging project is superior; Bai et al.^8^ merged topology theory with empirical data to develop a model for evaluating the grouting effect on water-rich sand layers, applying this model to assess the Qingdao subway grouting project. These methods effectively utilize data from grouting projects, integrating statistical analysis and the allocation of index weights to establish mathematical models for the quantitative evaluation of grouting effects, thereby yielding enhanced results in practical engineering applications, however, their reliance on the AHP method and empirical approaches can compromise the objectivity of these evaluations. Furthermore, advancements in grouting technology within the directional drilling sector bring about additional challenges in evaluating grouting effects due to increasingly complex geological and engineering scenarios. As machine learning algorithms continue to mature, an increasing number of engineering practices are incorporating them. Liu et al.^9^ utilized fuzzy neural networks, support vector machines, and random forest algorithms to develop a robust evaluation model for assessing the physical fitness of elderly individuals within the community, achieving satisfactory performance. In the evaluation of geologic disaster susceptibility in Li County, Sichuan Province, Zhou et al.^10^ identified 11 influential factors and employed both Random Forest and Radial Basis Function Neural Network algorithms to conduct landslide susceptibility assessments. These studies demonstrate that machine learning algorithms perform well under complex evaluation conditions and effectively address the challenges posed by complex and empirical factors in assessing grouting effects.

This paper focuses on the directional drilling and grouting transformation of gray rock aquifers in the Qiuji coal mine as the engineering context. It conducts a comprehensive analysis of the influencing factors affecting grouting effectiveness and establishes an evaluation system by integrating engineering parameters. Furthermore, it introduces eight machine learning models and three optimization algorithms. Building upon these foundations, a novel grouting effectiveness evaluation method tailored to the characteristics of directional drilling and grouting transformations in gray rock aquifers is proposed. This approach effectively addresses the challenges associated with complex factors, empirical observations, and extensive workload in grouting effectiveness assessments. The proposed method holds significant implications for the advancement of grouting evaluation techniques.

## Hydrogeologic overview of the study area

**Figure 1:**
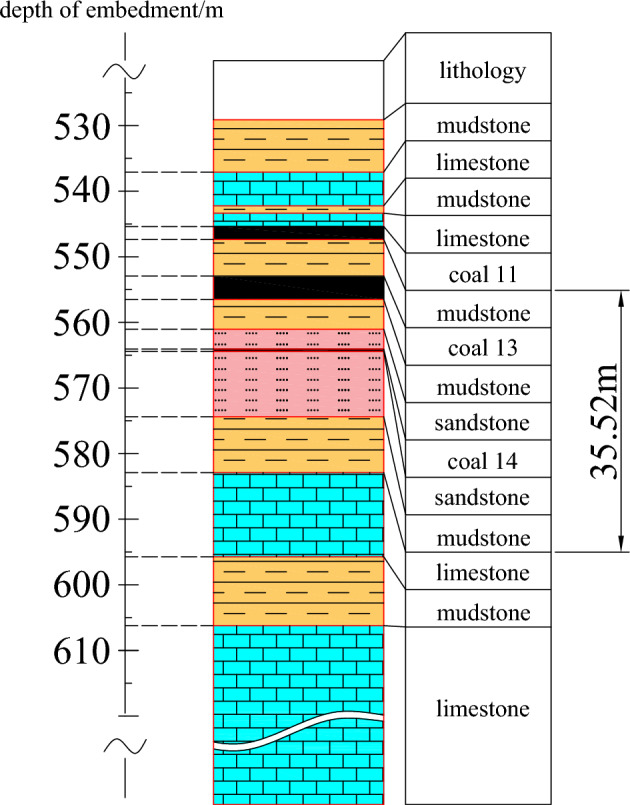
Stratigraphic Histogram.

The Qiuji Coal Mine, situated in Qihe County, Dezhou City, Shandong Province, represents the inaugural production mine in the Northern Yellow River Coalfield. The 11# and 13# coal seams within the mine are under threat from limestone aquifers, with Ordovician limestone aquifers present in its deeper sections, and these aquifers are hydraulically interconnected, adding to the mine's hydrogeological complexity, as shown in Fig. 1.

**Figure 2:**
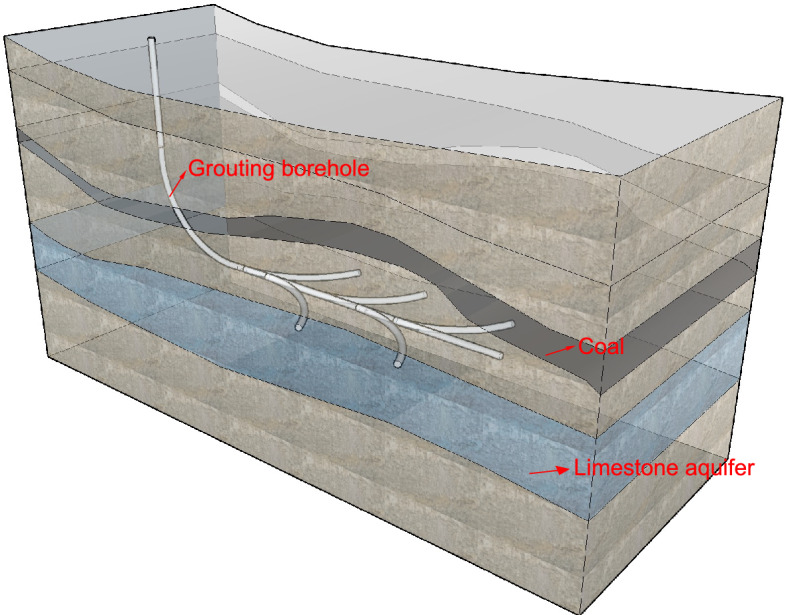
Schematic diagram of directional drilling grouting technology.

Qiuji Coal Mine initiated the use of directional drilling technology in 2016 (as shown in Fig. 2), segmenting the mining area into zones and utilizing near-horizontal branch-hole deployment for the grouting and transformation of limestone aquifers, effectively isolating the hydraulic connections between the aquifers, thereby ensuring safe coal resource production, and sustainable development of coal is realized.

## Analysis of factors and indicators influencing grouting effectiveness

### Main controlling factors of grouting effectiveness

#### Geological factors

1. Development of Karst fissures in tuffs.

**Figure 3:**
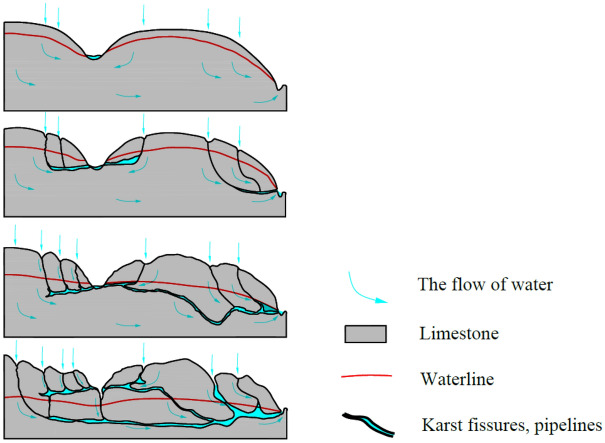
Schematic diagram of Karst fissure development process in limestone.

Limestone, a type of carbonate rock, is characterized by its solubility. Karst development in carbonate rocks begins with the dissolution of extended fissures, facilitated by erosive water flow, which continuously expands the fissure, eventually forming a dissolution pipeline. Once the dissolution pipeline widens to a certain extent, the water flow’s energy becomes sufficient to transport silt and sand, intensifying the mechanical erosion of the pipeline and thus becoming the primary force behind cavern expansion. When the cavern expands to a certain size, it collapses under the action of gravitational forces from the overlying load, making gravity-induced collapse the main driver of this transformation. Therefore, the transformation and evolution of the karst water system can be summarized as a progression from dissolution to mechanical erosion, followed by gravity-induced collapse, as illustrated in Fig. 3.

Influenced by a range of factors including the lithology of rock strata, tectonic activity, and climate, karst development in China displays distinct regional differences between the north and south: the southern karst regions show extraordinary development, with widespread occurrences of dissolution, mechanical erosion, and gravity collapse, and a prevalent underground river system; whereas in the north, karst development is relatively weaker, predominantly characterized by dissolution and mechanical erosion, with dissolution fissures or pipeline-caves being more common, and underground dark rivers being exceedingly rare^11^.

Karst fissure development influences groundwater storage and flow, as well as the diffusion of grout in the medium. Although limestone bedrock is dense and hard, it is characterized by numerous dissolution fissures and pipes, leading to the grouting in limestone aquifers predominantly exhibiting characteristics of flowing along these fissures. Thus, the development of limestone karst fissures directly impacts the diffusion of slurry and the effectiveness of grouting transformation.

2. Geological structure.

**Figure 4:**
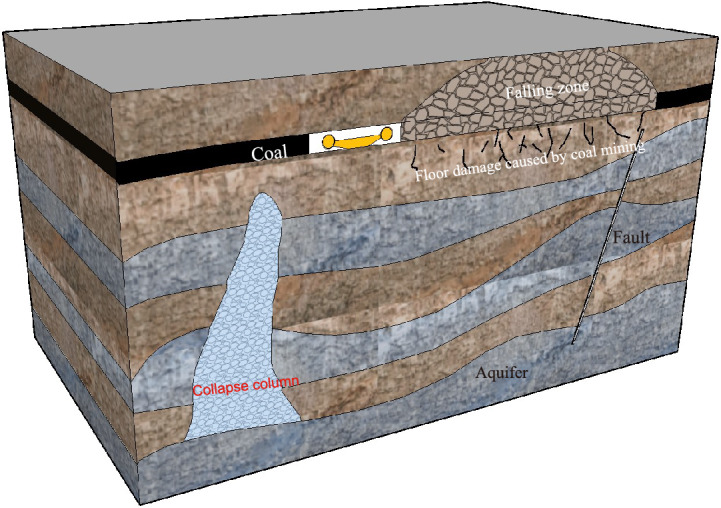
Schematic diagram of the construction.

In the process of excavating deep roadways, crossing geological structures like fault fragmentation zones, and trapped columns is inevitable, as depicted in Fig. 4. These geological formations are characterized by their proneness to deformation, high water permeability, low strength, and poor water resistance, making them susceptible to becoming water-conducting channels, potentially leading to water-surge phenomena^12–15^. Additionally, these features facilitate the spread of slurry. Consequently, various factors, including the spatial spreading pattern and characteristics of these geological structures, significantly influence the effectiveness of grouting transformation^16^.

3. Water-bearing conditions of the limestone aquifer.

**Figure 5:**
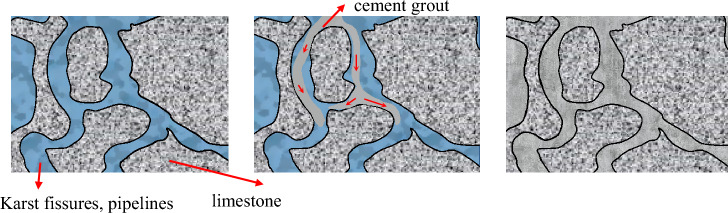
Schematic of karst water slurry replacement process.

Karst fissures within limestone aquifers typically exhibit water saturation, and grouting modification in limestone aquifers essentially involves the substitution of slurry for water within these karst fissures^17^, as illustrated in Fig. 5. Consequently, the initial water volume within the karst fissures of a limestone aquifer directly influences the extent of grouting required.

4. Alterations in aquifer permeability characteristics.

The purpose of aquifer grouting is to reduce mine water surges. According to some scholars, the permeability of geological strata is a critical factor in determining the likelihood of water burst events^18^. Modifying a limestone aquifer with grouting is expected to significantly change its permeability characteristics, making changes in permeability a key indicator of grouting effectiveness.

#### Factors influencing engineering outcomes

1. Drilling work quality.

For the grouting transformation of limestone aquifers, it is imperative to construct directionally oriented down-layer branch holes to investigate the aquifer prior to the grouting process. This step is crucial to maximize the exposure of karst fissures and unique geological structures within the limestone aquifer. The primary objective is to ensure extensive horizontal drilling along the aquifer, followed by the secondary goal of maintaining the integrity of the drill holes. This approach aims to prevent issues such as the collapse or blockage of the drill holes. Consequently, high-quality drilling is paramount to ensuring the efficacy of the grouting transformation process^19^.

2. Slurry diffusion and transmission scope.

**Figure 6:**
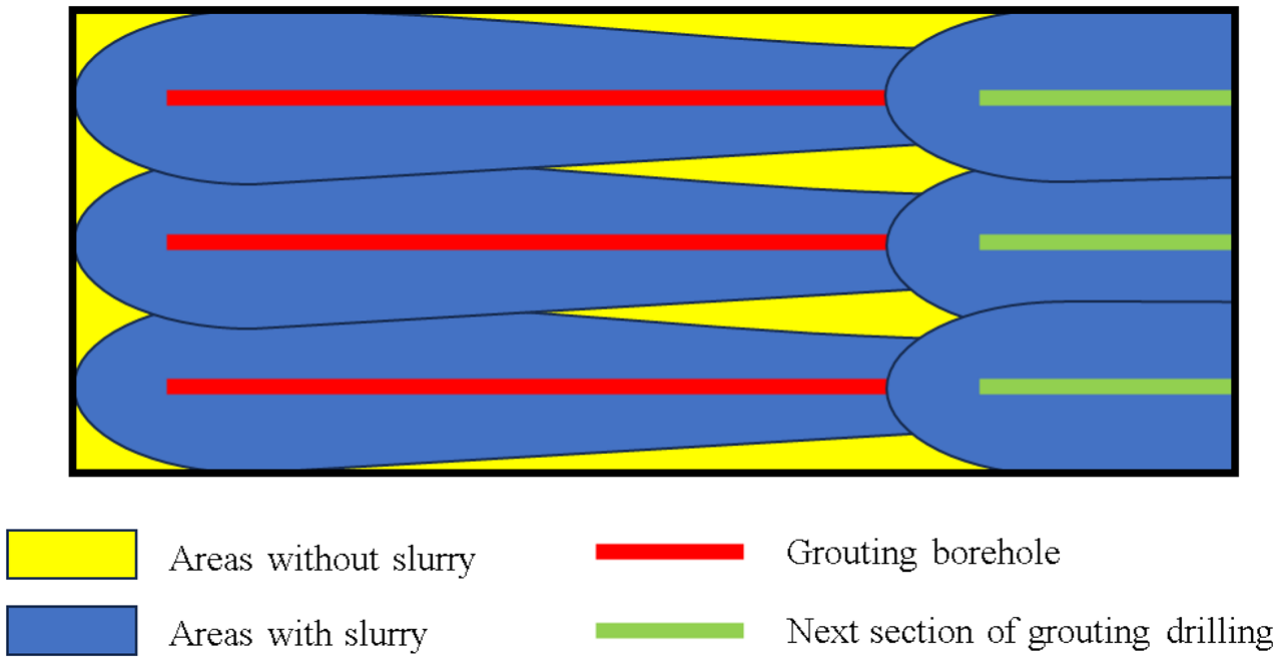
Slurry diffusion.

The limestone aquifer grouting renovation project utilizes horizontal branch hole segmental grouting technology. The arrangement of grouting holes follows a parallel branch hole pattern in horizontal segments. This approach is characterized by extended conveying and grouting sections, predominantly employing high-pressure grouting techniques. The transmission of the slurry within the horizontal holes influences its effective dispersion range, consequently impacting the overall efficacy of the grouting process. Furthermore, the extent to which the slurry's dispersion encompasses the entire grouting transformation area and the presence of any grouting blind zones are indicative of the grouting's effectiveness, as depicted in Fig. 6.

3. Fissure filling processes in Karst environments.

The limestone aquifer contains numerous karst fissures, which are extensively water-filled. Fundamentally, the grouting transformation of the limestone aquifer involves replacing the water within these fissures with slurry. This replacement significantly reduces the aquifer’s water content and hydraulic conductivity. Consequently, the efficacy of grouting transformation is directly contingent upon the effective filling of these karst fissures.

### Analysis of grouting effectiveness parameters

#### Drilling operation parameters

1. The proportion of boreholes in the target rock strata.

The proportion of drill holes reaching the target rock layer directly indicates the effectiveness of horizontal layer drilling, thereby reflecting the overall quality of the drilling project. According to the technical quality indices for ground drilling construction, in geological exploration, the proportion of drill holes in the target rock layer should be no less than 90%.

2. Drilling length.

As the horizontal drilling length increases during the grouting process, the slurry flow encounters resistance due to factors such as drilling wall friction and fissure diversion, consequently, the effective grouting pressure diminishes progressively with the drilling hole length. Additionally, excessive drilling hole length leads to greater head loss of the slurry, resulting in insufficient effective grouting pressure, a limited slurry transfer range, the presence of grouting blind zones, and ultimately, a suboptimal grouting renovation effect.

3. Leakage.

During the drilling process, substantial variations are observed in the consumption of drilling fluid when exposing water-conducting channels of varying developmental extents^20^. Consequently, the volume of drilling fluid leakage serves as a crucial indicator of the extent of karst fissure and tectonic development within limestone aquifers.

4. Water level.

During the drilling process, consistent monitoring of the water level at 30-min intervals is mandated prior to grouting, until stabilization is confirmed through three successive observations, preceding any further operations. Water level elevation refers to the height indicated by the pressurized water within the pressurized aquifer, as it rises within the borehole, and this measurement can to a certain extent reflect the water content of the limestone aquifer in the vicinity of the borehole.

#### Grouting engineering parameters

1. Pressure of grouting.

Grouting pressure serves as the primary driving force enabling the slurry to penetrate karst fissures via horizontal drilling holes. Consequently, grouting pressure directly influences the slurry's transportation within the horizontal drilling hole and karst fissure, thereby impacting the slurry's ability to fill these fissures^21^. On one hand, grouting pressure is transformed into the kinetic energy of the slurry, while on the other, it is gradually dissipated due to various factors including friction between the slurry and the borehole wall, directional changes as the slurry enters the karst fissures, and the intrinsic properties of the slurry itself.

2. Mass of slurry injected.

The quality of the injected slurry can serve as an indicator to a certain extent for evaluating the grouting effect^22^, however, the correlation between the quality of injected slurry and the grouting effect remains ambiguous. The quality of injected slurry is significantly influenced by the geological conditions of the grouting area, and directly evaluating the grouting effect based solely on the quality of injected slurry proves challenging. Thus, it is often necessary to incorporate a range of parameters in the assessment.

3. Alterations in the permeability characteristics of limestone aquifers pre- and post-grouting.

The principal indicators of rock body permeability are the water absorption rate and the permeability coefficient^23^. Water absorption rate represents a physical measure of the extent of water absorption under standard atmospheric pressure. In grouting projects, water pressure tests are conducted both before and after grouting. The pre-grouting water pressure test aids in preliminarily assessing the development of karst fissures, potential connections between adjacent aquifers, and the presence of geological structures like faults and trap columns. Post-grouting water pressure tests are utilized to evaluate the slurry's filling effectiveness in fissures and structures. The permeability coefficient is a measure indicating the difficulty for fluids to traverse the pore skeleton; a lower coefficient signifies greater difficulty for fluid passage. Generally, limestone aquifers have a very low permeability coefficient; however, the presence of karst fissures results in a relatively higher coefficient at the macroscopic level. Grouting modifications can significantly reduce this coefficient. The formulae for both are shown in Eqs. (1)and(2).

1 $$q = \frac{Q}{HL}$$

where: Q for the pressure into the flow, L/min; H for the role of the test section of the pressure (converted head height); L for the injection section length, m.

2 $${\text{ K}} = \frac{T}{2\pi HL}ln\frac{L}{r}$$

where: T is the flow rate of pressurized water, m^3^/d; r is the radius of the borehole, m.

### Relationship between parameters and influencing factors

#### “Dual-process, multi-parameter, and multi-factor” system

**Figure 7:**
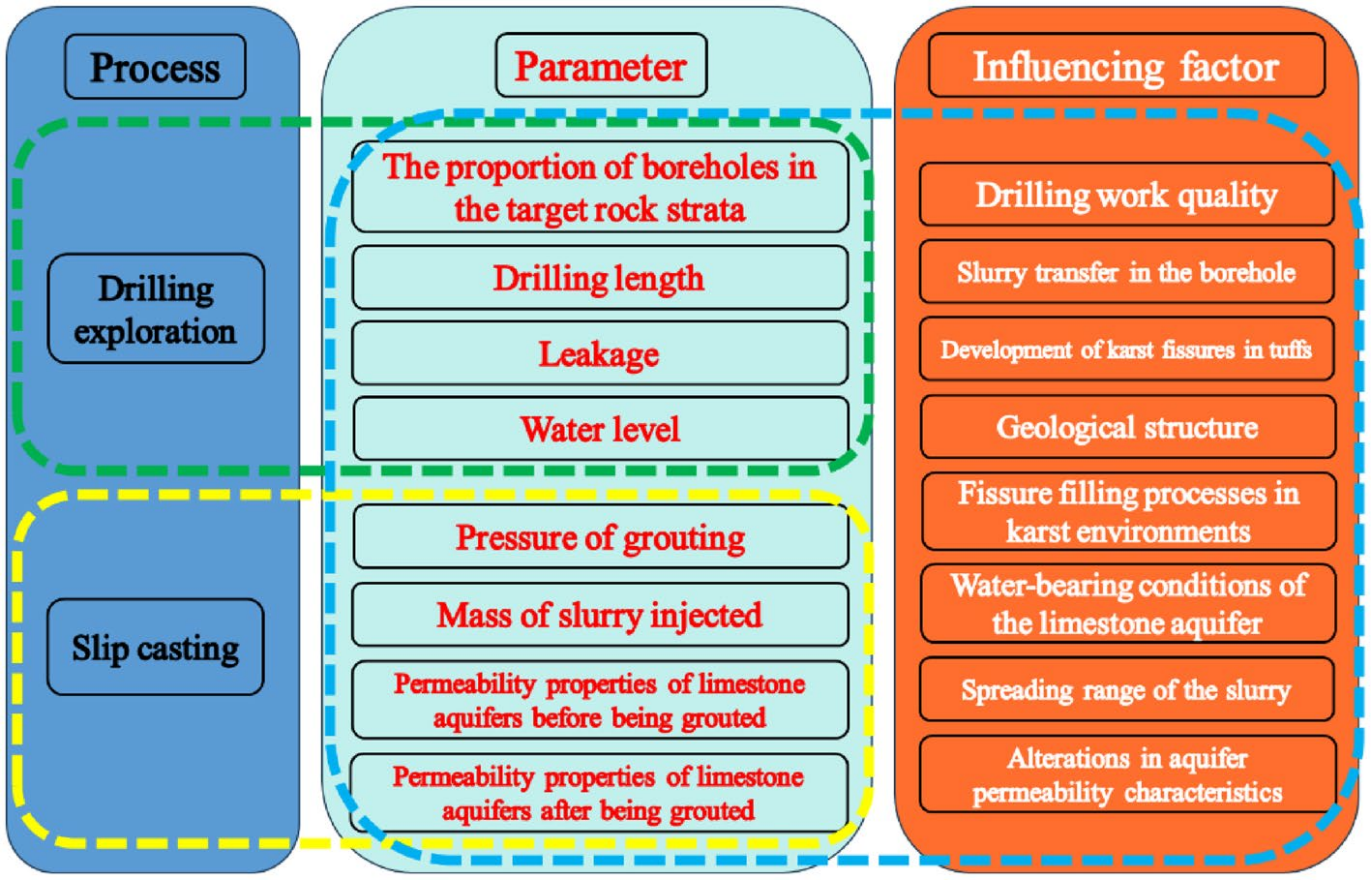
“Dual-process, multi-parameter, and multi-factor” system.

Parameters within distinct processes are indicative of various influencing factors, and frequently, a single influencing factor may be represented by several parameters collectively. Considering the two processes of drilling and grouting, four parameters from each process were gathered. This resulted in a total of eight parameters: the proportion of boreholes in the target rock strata, drilling length, leakage, water level, pressure of grouting, mass of slurry injected, permeability properties of limestone aquifers before being grouted, permeability properties of limestone aquifers after being grouted, which collectively cross-referenced the eight factors, thereby establishing a multi-parameter system that reflects these multifaceted factors, as illustrated in Fig. 7.

#### Examination of the interrelation among indicators

Pearson correlation coefficient is used to measure the degree of linear correlation between the data and its expression is shown in Eq. (3)^24^.

3 $$\rho_{x,y} = \frac{{\sum\limits_{i = 1}^{n} {\left( {X_{i} - \overline{X} } \right)} \left( {Y_{i} - \overline{Y} } \right)}}{{\sqrt {\sum\limits_{i = 1}^{n} {\left( {X_{i} - \overline{X} } \right)}^{2} } \sqrt {\sum\limits_{i = 1}^{n} {\left( {Y_{i} - \overline{Y} } \right)}^{2} } }}$$

where: (*X*_*i*_*, Y*_*i*_) is the *i* th value of any two sets X, Y; $\left( {\overline{X} } \right.$, $\left. {\overline{Y} } \right)$ is the mean value of the two sets; *n* is the number of set elements.

**Figure 8:**
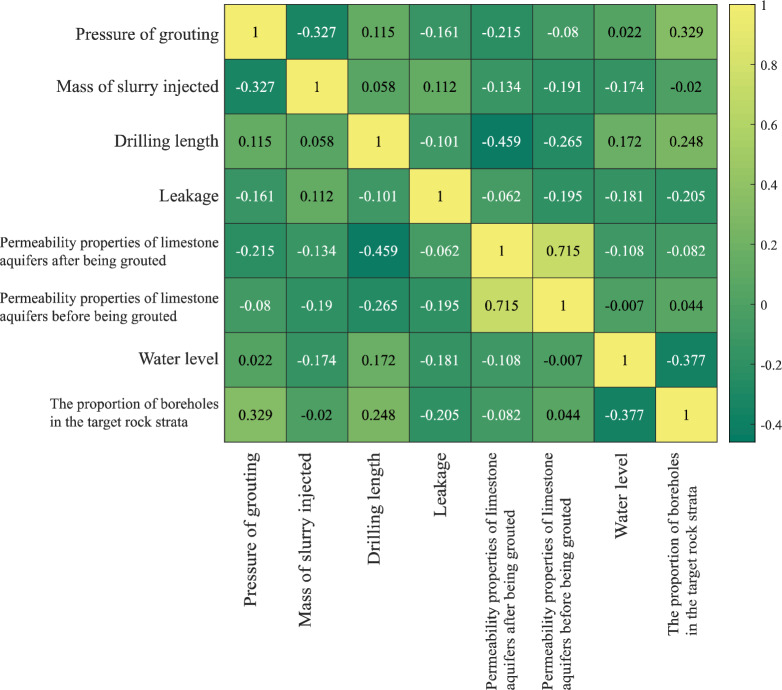
Correlation analysis.

**Figure 9:**
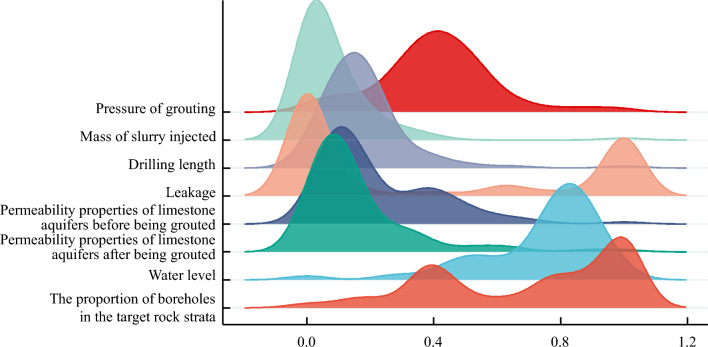
Distribution of data by indicator.

**Figure 10:**
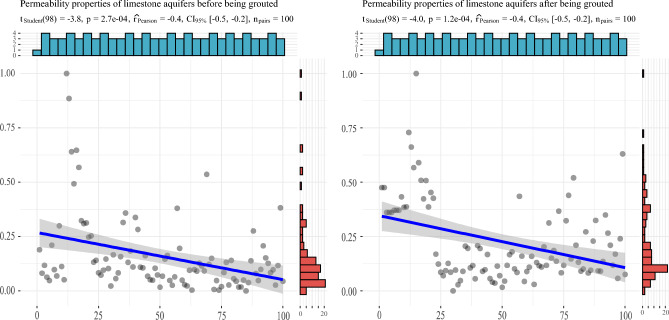
Correlation analysis of permeability properties of limestone aquifer before and after grouting.

**Figure 11:**
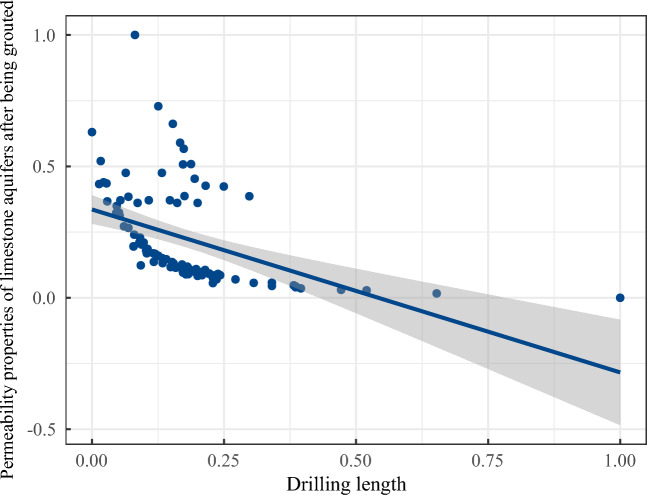
Correlation analysis between infiltration performance and borehole section length after grouting.

The analysis results are depicted in Figs. 8, 9, 10 and 11. There is some similarity in the distribution of data for individual indicators (see Fig. 9). The permeability performance of the limestone aquifer, both pre- and post-grouting, represents varying conditions of the same index, with a correlation of 0.715, indicating moderate correlation (Fig. 10). Concurrently, it was observed that the post-grouting permeability performance has a correlation of 0.459 with the length of the grouting section, indicative of a low correlation (Fig. 11). The primary reason for this is that the permeability performance is determined through compression testing, where the length of the drilled section plays a crucial role as a key physical parameter.

## Modeling and evaluation

### Model building process

**Figure 12:**
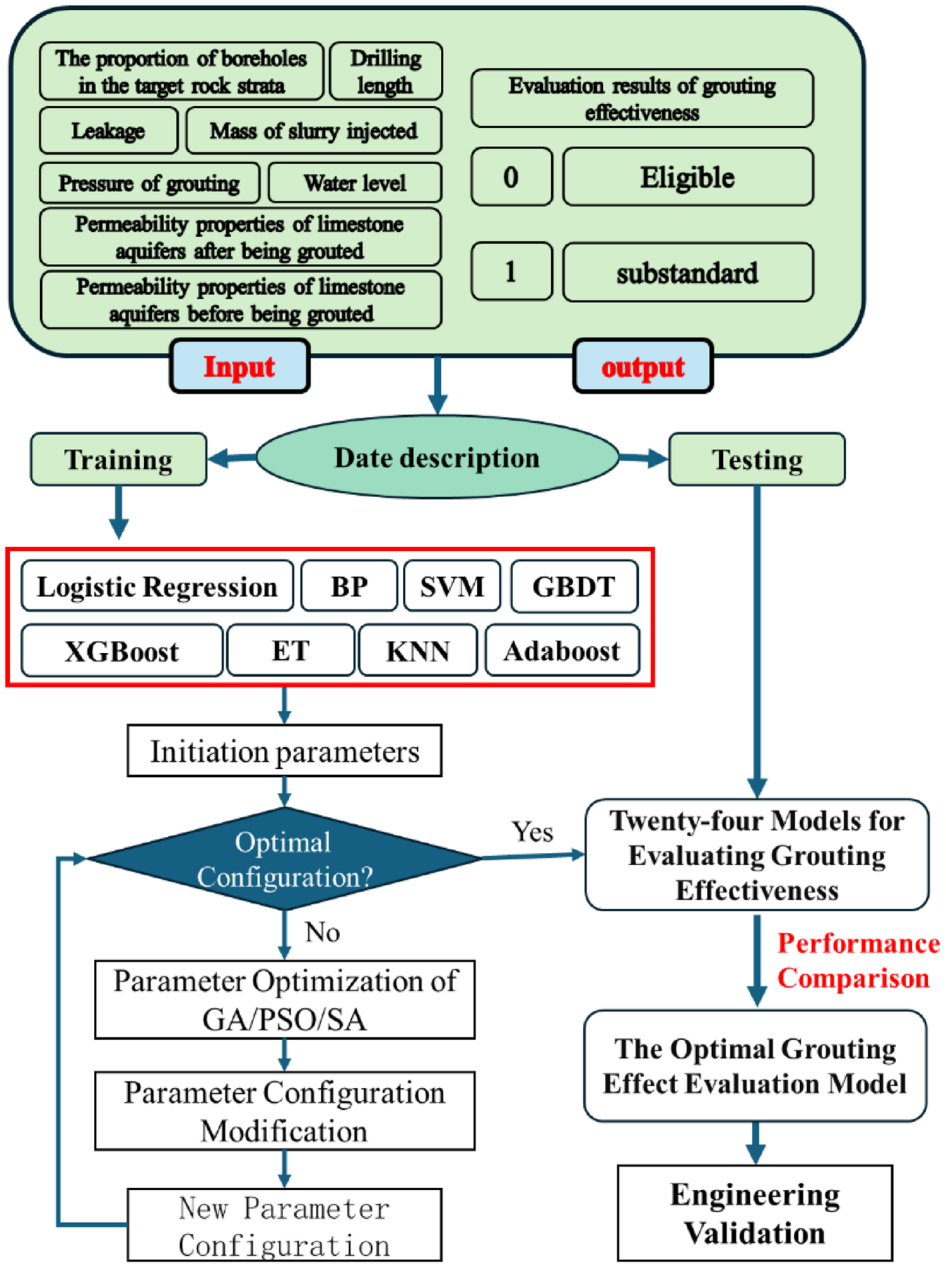
Modeling process for evaluating grouting effects using machine learning algorithms.

To elucidate the model building and evaluation process, a flowchart (Fig. 12) is employed. The specific steps are outlined below:

Step 1: Compile the evaluation model dataset using field-recorded geological and grouting engineering data, incorporating indicators with actual evaluation outcomes.

Step 2: Preprocess the data and partition it into training and testing sets.

Step 3: Train the machine learning models using the training data set.

Step 4: Optimize the machine model parameters to determine the best configuration for each machine learning algorithm, resulting in 24 grouting effect evaluation models.

Step 5: Compare the performance of the 24 grouting effect evaluation models using the test data set to identify the optimal evaluation model.

Step 6: Verify the model through engineering practice.

### Machine learning model

We introduced eight machine learning models: Logistic Regression, Extreme Gradient Boosting (XGBoost), Support Vector Machine (SVM), K-Nearest Neighbor (KNN), Gradient Boosted Decision Tree (GBDT), Extremely Randomized Trees (ET), BP Neural Network, and Adaptive Boosting (AdaBoost), all of which demonstrate superior performance.

Among these, Logistic Regression is a classification algorithm relying on linear regression, primarily utilized for binary classification tasks; KNN is an instance-based learning algorithm that predicts by identifying the nearest point within the training data; SVM classifies by constructing an optimal hyperplane; XGBoost and Adaboost are both ensemble learning algorithms that assemble a robust classifier by aggregating multiple weak classifiers to enhance model performance; GBDT and ET are both ensemble learning algorithms rooted in decision trees; GBDT trains decision trees iteratively, adjusting sample weights based on previous prediction outcomes in each iteration to prioritize mispredicted samples in subsequent trees; in contrast, ET trains each tree through random feature divisions, enhancing model diversity; BP represents an artificial neural network that adjusts network weights and biases by computing the error between predicted and true values and propagating the error backward throughout the network, progressively aligning network output with true values.

### Enhancing model efficacy through parameter optimization

In machine learning, the configuration of model parameters is a critical factor influencing model performance. To ensure the rationality and scientific basis of parameter settings, heuristic optimization algorithms are employed. Diverse machine learning models exhibit varying degrees of adaptability to different optimization algorithms. Consequently, three distinct algorithms: Genetic Algorithm, Particle Swarm Optimization, and Simulated Annealing are utilized. This approach facilitates the optimization of various parameters across different models.

The Genetic Algorithm is an optimization method grounded in evolutionary theory. It perpetuates genetic information across generations through a series of operations like replication, mutation, and crossover, employing the principle of survival of the fittest to retain highly adaptive results, ultimately converging to the optimal solution^25^. This algorithm's advantages include robust search capabilities and rapid computation, though it is prone to convergence on local optima. The optimization process for each parameter of the machine learning model involves several steps: setting the population size based on the number of parameters, evaluating fitness, selecting parent individuals based on fitness values, performing crossover operations on selected parents to produce offspring, applying mutation operations to offspring by randomly altering their genes, combining parent and offspring individuals to create the next generation of the population, and iterating these steps until optimal parameter configurations are achieved.

The Particle Swarm Optimization algorithm, an evolutionary computation technique, is inspired by the collective foraging behavior of birds in nature. This approach is extensively utilized in engineering due to its simple implementation, efficient search capability, and rapid convergence^26^. The fundamental concept of PSO is that each particle in the swarm benefits from the collective experience and knowledge accumulated by all members, facilitating the search for the optimal solution through collaboration and information sharing among the particles^27^. PSO algorithms are also susceptible to convergence on local optima. The optimization process involves several steps: determining the number of particle swarms based on the number of parameters, initializing velocity and position vectors for each particle, assessing particle performance in the problem space, updating particle velocity and position based on their historical best positions and the historical best positions of the entire swarm, and iterating these steps to attain the optimal solution.

Simulated Annealing algorithms can address complex problems and surmounting local minima in the optimization process. Annealing involves heating a solid to a temperature high enough for molecules to become randomly aligned, followed by gradual cooling, resulting in molecules aligning in a low-energy state^28^. The primary advantage of this algorithm is its exhaustive search capability, reducing the likelihood of settling on a local optimum. However, it has the drawbacks of slow convergence and extended execution time. The optimization process involves several steps: randomly generating initial parameters, setting the initial temperature and cooling rate, perturbing the current parameter configuration to obtain a new candidate solution, evaluating the performance of the candidate solution, calculating the acceptance probability based on the performance of the current and new solutions, deciding whether to accept the new solution, adjusting the temperature according to the cooling rate, and iterating these steps to achieve the optimal solution.Table 1 displays the parameter settings for the three optimization algorithms.

**Table 1:** Optimization algorithm parameter settings.

Algorithm	Parameter	Setting	Range of values
GA	Population size	100	20–150
	Maximum Iterations	300	100–500
	Mutation rate	0.01	0.0001–0.1
	Crossover rate	0.5	0.4–0.99
PSO	Swarm size	100	20–150
	Maximum Iterations	300	100–500
	Inertia weight	0.9	0.6–1.2
	Cognitive component	2	0–4
	Social component	2	0–4
SA	Initial temperature	100	100–200
	termination temperature	2	1–5
	Cooling rate	0.98	0.95–0.99
	Number of iterations	200	100–200

### Evaluation metrics for model performance

1. Confusion matrix.

**Figure 13:**
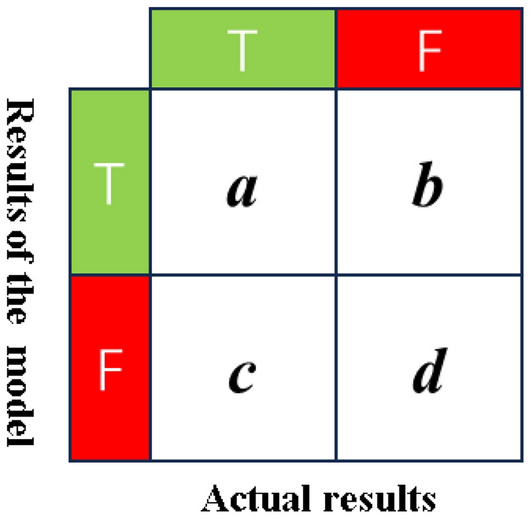
Confusion matrix.

In predictive and classification models, the confusion matrix serves as a visual representation of the model’s performance^29^. Assuming a model evaluates M samples with binary classification outcomes denoted as T (True) and F (False), the confusion matrix is illustrated in Fig. 13. Here, “a” represents the count of samples predicted as T and actual as T, “b” as predicted T but actual F, “c” as predicted F but actual T, and “d” as both predicted and actual F, with the total sample count M equal to a + b + c + d.

2. Accuracy.

**Figure 14:**
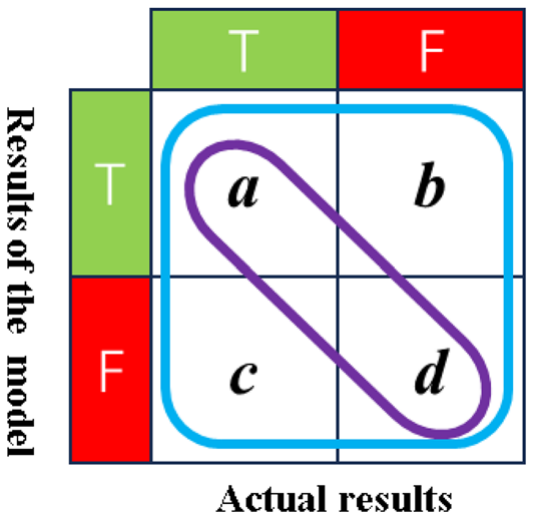
Confusion matrix for accuracy.

Accuracy denotes the ratio of correctly predicted samples to the total number of samples in the modeling outcomes, with higher accuracy generally indicative of superior model performance. As illustrated in Fig. 14, the formula for calculating accuracy is as follows:

4 $$A = \frac{a + d}{M}$$

3. Recall.

**Figure 15:**
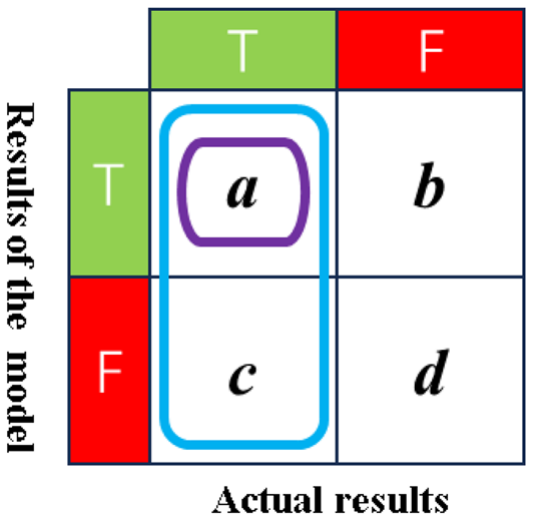
Confusion matrix for recall.

Recall, pertaining to actual outcomes, measures the proportion of samples that are truly classified as T and are correctly predicted as T, as depicted in Fig. 15. The formula for its calculation is as follows:

5 $$R = \frac{a}{a + c}$$

4. Precision.

**Figure 16:**
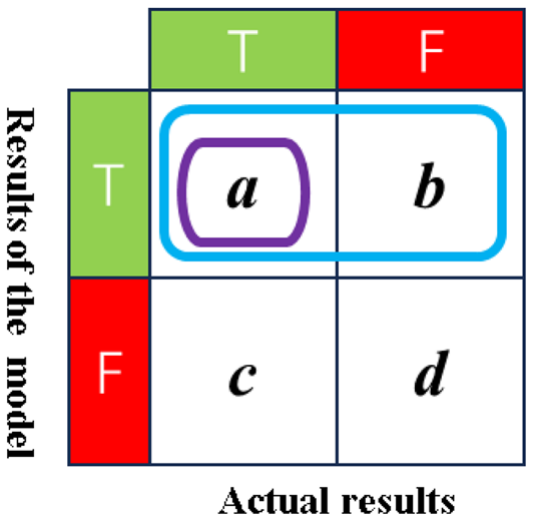
Confusion matrix for precision.

Precision, concerning predicted outcomes, quantifies the proportion of samples that are predicted as T and are indeed correctly classified as T, as depicted in Fig. 16.

6 $$P = \frac{a}{a + b}$$

5. F.

The F-score, a harmonizing metric, is introduced to equilibrate recall and precision^30^. It is generally computed using the following equation:

7 $$F = \frac{2PR}{{P + R}}$$

### Evaluation of model performance

**Figure 17:**
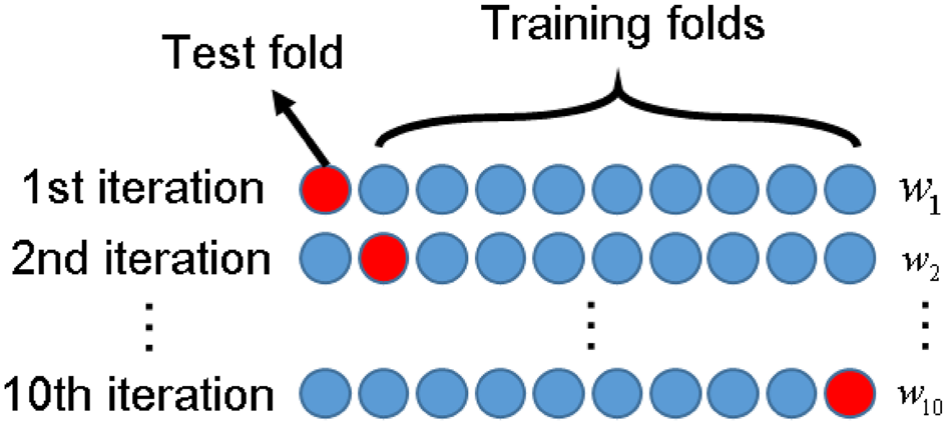
A sketch map for tenfold cross validation.

To comprehensively measure the performance of the model, indicators including accuracy, recall, precision, and F-score were introduced. The evaluation was conducted by investigating the pre-grouting project at Qiuji coal mine, where 100 sets of data from grouting projects were collected (As in the supplementary material). The model's predictive performance was assessed using the tenfold cross-validation method. This method, illustrated in Fig. 17, involves obtaining 10 mutually exclusive subsets of similar sizes through stratified sampling of the dataset. Each subset serves as a test set, while the remaining 9 subsets are utilized as training sets. Subsequently, after 10 training iterations, the average of the results from the 10 test sets is computed to evaluate the model’s performance, as shown in Eq. (8).

8 $$w = \frac{1}{10}\sum\limits_{i = 1}^{10} {w_{i} }$$

**Figure 18:**
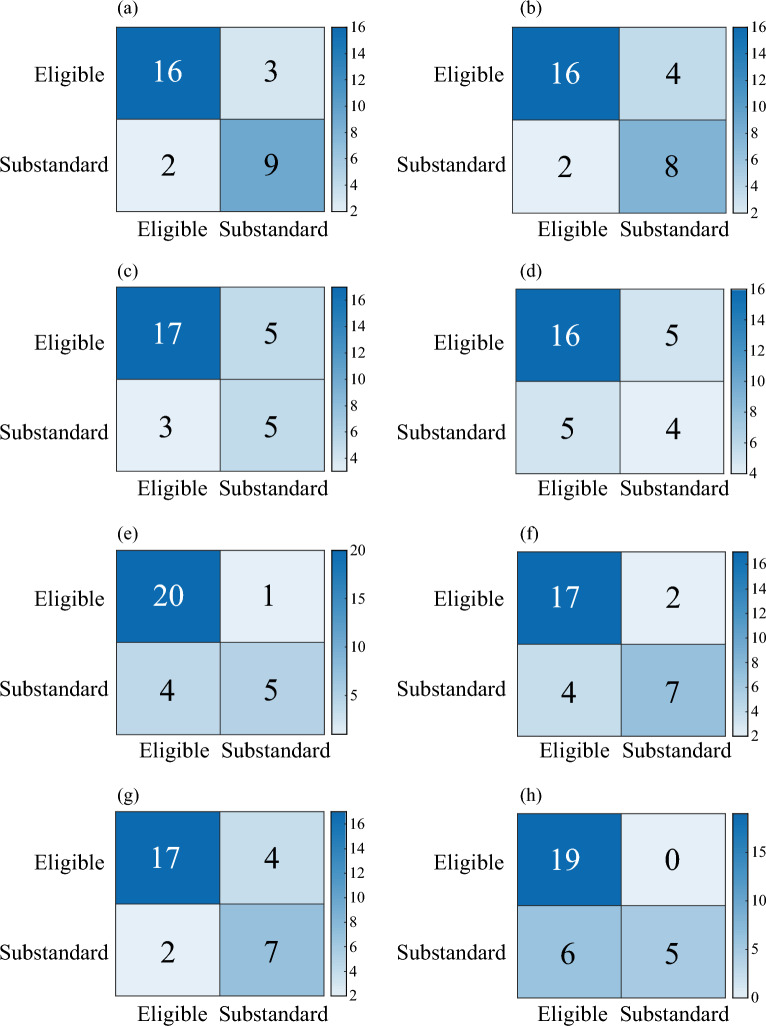
Confusion matrix of machine learning model test set: (**a**) Adaboost; (**b**) BP; (**c**) ET; (**d**) GBDT; (**e**) KNN; (**f**) LR; (**g**) SVM; (**h**) XGBoost.

Different heuristic optimization algorithms were employed to optimize the model parameters. The calculated accuracy, recall, precision, and F-score of the eight grouting effect evaluation models are summarized in Table 2. Additionally, the confusion matrix of the test set for each of the eight evaluation models is depicted in Fig. 18.

**Table 2:** Results of machine learning.

Model		BP		Adaboost		GBDT		ET	
		Training	Test	Training	Test	Training	Test	Training	Test
GA	Accuracy	0.73	0.80	0.87	0.83	0.76	0.63	0.90	0.77
	Recall	0.73	0.80	0.87	0.83	0.76	0.63	0.90	0.77
	Precision	0.73	0.85	0.88	0.84	0.77	0.69	0.91	0.84
	F	0.72	0.78	0.87	0.84	0.73	0.59	0.90	0.74
PSO	Accuracy	0.69	0.73	0.67	0.83	0.99	0.67	1.00	0.73
	Recall	0.69	0.73	0.67	0.83	0.99	0.67	1.00	0.73
	Precision	0.68	0.73	0.67	0.84	0.99	0.66	1.00	0.74
	F	0.68	0.73	0.65	0.82	0.99	0.66	1.00	0.72
SA	Accuracy	0.69	0.80	0.94	0.73	0.89	0.67	0.94	0.73
	Recall	0.69	0.80	0.94	0.73	0.89	0.67	0.94	0.73
	Precision	0.70	0.82	0.94	0.73	0.90	0.67	0.94	0.76
	F	0.69	0.80	0.94	0.73	0.88	0.67	0.94	0.74
/	Accuracy	0.76	0.60	1.00	0.63	0.59	0.67	1.00	0.73
	Recall	0.76	0.60	1.00	0.63	0.59	0.67	1.00	0.73
	Precision	0.80	0.63	1.00	0.68	0.34	0.44	1.00	0.77
	F	0.73	0.52	1.00	0.65	0.43	0.53	1.00	0.71

Model		LR		SVM		Xgboost		KNN	
		Training	Test	Training	Test	Training	Test	Training	Test
GA	Accuracy	0.69	0.80	0.71	0.77	/	/	0.67	0.77
	Recall	0.69	0.80	0.71	0.77	/	/	0.67	0.77
	Precision	0.69	0.80	0.72	0.78	/	/	0.67	0.76
	F	0.66	0.80	0.71	0.77	/	/	0.66	0.76
PSO	Accuracy	0.74	0.77	0.71	0.73	1.00	0.73	0.74	0.73
	Recall	0.74	0.77	0.71	0.73	1.00	0.73	0.74	0.73
	Precision	0.75	0.77	0.71	0.83	1.00	0.73	0.75	0.72
	F	0.73	0.76	0.71	0.75	1.00	0.73	0.73	0.72
SA	Accuracy	0.73	0.80	0.76	0.80	0.90	0.80	0.77	0.83
	Recall	0.73	0.80	0.76	0.80	0.90	0.80	0.77	0.83
	Precision	0.73	0.81	0.76	0.82	0.91	0.85	0.84	0.83
	F	0.71	0.79	0.76	0.81	0.90	0.78	0.75	0.82
/	Accuracy	0.71	0.70	0.73	0.67	1.00	0.60	0.79	0.70
	Recall	0.71	0.70	0.73	0.67	1.00	0.60	0.79	0.70
	Precision	0.71	0.80	0.77	0.60	1.00	0.59	0.80	0.71
	F	0.71	0.64	0.71	0.63	1.00	0.59	0.78	0.70

Results indicate that parameter optimization via heuristic algorithms enhances the evaluation model’s performance, evidenced by an increase in the average F-score from 0.71 pre-optimization to 0.77 post-optimization. Additionally, the selection of heuristic algorithms significantly impacts the model's final performance, with varying optimization effects observed across different algorithms. The model results, using the test set's F-score as a performance metric, reveal that the best performing models include BP Artificial Neural Network (0.80), AdaBoost (0.84), Logistic Regression (0.80), KNN (0.82), and SVM (0.81), with the AdaBoost model, optimized via a genetic algorithm, exhibiting superior performance. Conversely, EXTRA TREE (0.74), GBDT (0.67), and XGBoost (0.78) demonstrated relatively lower performance, potentially attributable to the limited dataset size impacting model training.

## Comparison and validation of actual projects

**Figure 19:**
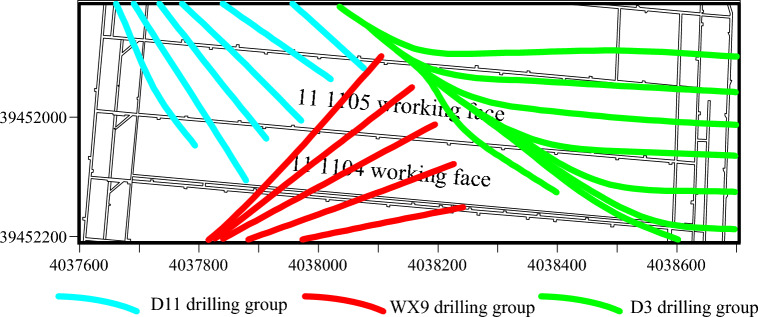
Arrangement of drill holes.

**Figure 20:**
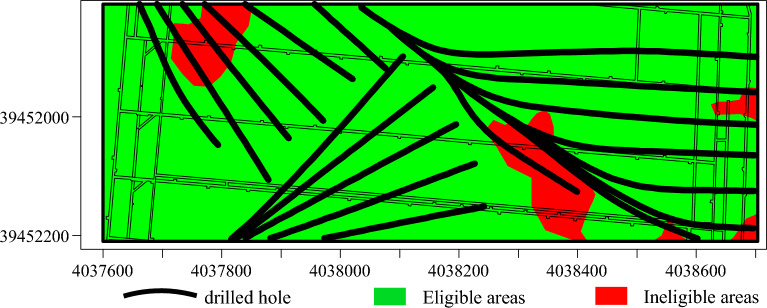
Assessment outcomes of traditional grouting effectiveness evaluation techniques.

The 11 1104 and 111105 working faces of Qiuji Coal Mine, situated in mining district 11, feature elevations ranging from − 425 to – 340 m. The 1104 working face spans a width of 100 m, while the 1105 working face extends across 120 m. The Xu ash limestone aquifer, located beneath the working face, has an average thickness of 12.85m and serves as an indirectly water-filled aquifer for the mining of the 11 coal seams. Borehole pumping test data reveal that the Xu ash aquifer’s water influx ranges from 0.286 to 1.658 L/s m, with medium to strong water richness. The permeability coefficient varies from 0.0169 to 6.2225 m/d, reducing to 0.2296 m/d after grouting modification. The water chemistry is categorized as SO^4-^Ca, SO^4-^Ca∙Mg, HCO^3^∙SO^4-^Ca∙Mg, with mineralization levels of 0.438–3.130 g/L. To ensure safe mining operations, ground area directional grouting technology was employed to treat the Xuhui aquifer. The ground drilling operations near the two working faces involved drilling groups D3, D11, and WX9. The arrangement of these ground drilling holes is depicted in Fig. 19. Following the grouting transformation, as illustrated in Fig. 19, the grouting effect was evaluated using traditional methods. The results of this evaluation are presented in Fig. 20.

**Figure 21:**
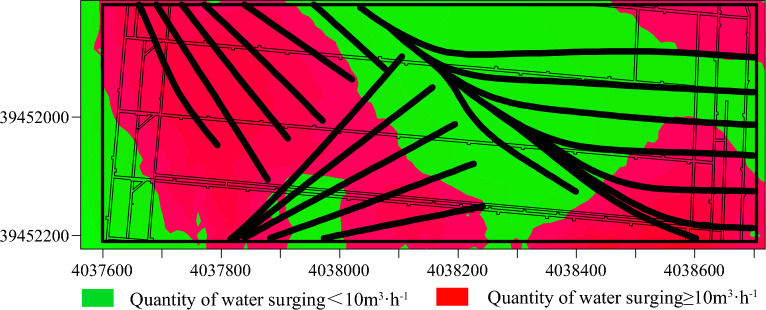
Surge zoning.

Following the grouting treatment, there has been a substantial reduction in the water richness of the Xu Lime limestone aquifer. However, mine borehole explorations have verified the presence of areas with significant water influx (≥ 10 m^3^/h), suggesting suboptimal grouting effectiveness in these regions, as illustrated in Fig. 21.

**Figure 22:**
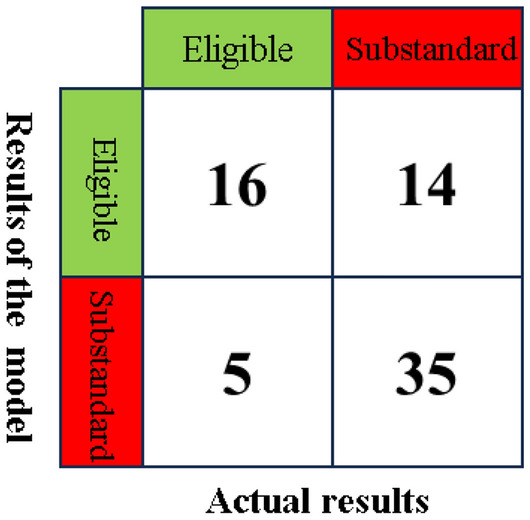
Confusion matrix for evaluation results.

Using SPSS PRO software, the engineering data from borehole groups D3, D11, and WX9 were imported into the model to assess the grouting effect using a trained AdaBoost machine learning model optimized by genetic algorithms. The evaluation results are depicted in Fig. 22. The overall accuracy rate of the model is 72.9%. For the attainment case, the model's recall rate is 76.2%, while the precision rate is only 53.3%, indicating insufficient training for the attainment case and more misclassification of non-attainment cases as attainment. This issue may be attributed to the limited amount of data for the attainment case in the dataset. It is expected that increasing the data volume will improve the situation. The F-score is 61.8%, indicating an overall passing grade. For the non-attainment case, the model’s recall rate is 61.8%, also a passing grade overall. Additionally, for the non-attainment case, the model’s recall rate is 72.9%. In this case, the recall rate is 71.4%, the precision rate is 87.5%, and the F-score is 78.6%, indicating higher evaluation performance.

**Figure 23:**
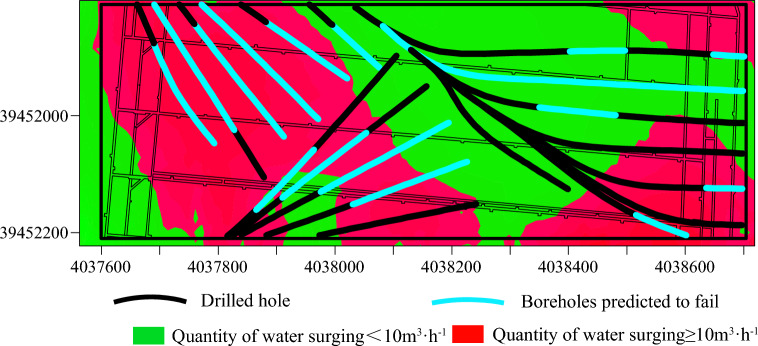
Predicted substandard boreholes.

**Figure 24:**
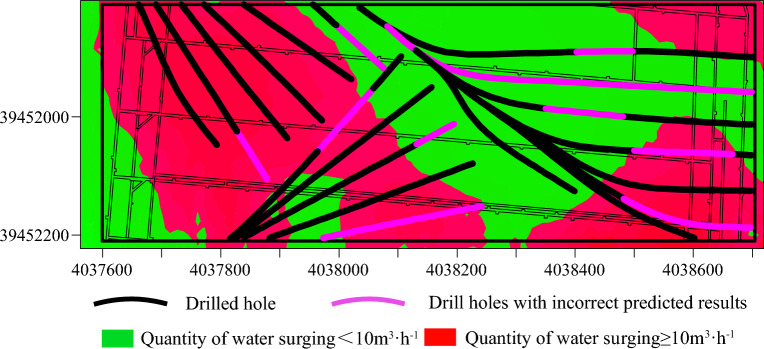
Incorrectly predicted boreholes.

Figure 23 displays the boreholes predicted to exhibit substandard grouting effects. These boreholes are primarily situated in areas of higher water influx, suggesting a significant correlation between the prediction results and the actual conditions. The boreholes with inaccurate predictions are illustrated in Fig. 24.

A comparative analysis of Figs. 20 and 21 reveals that the Adaboost grouting effect evaluation model, optimized by a genetic algorithm, demonstrates higher accuracy compared to traditional evaluation methods and aligns more closely with the actual outcomes of physical exploration, thereby suggesting that the GA-Adaboost model serves as a viable reference for evaluating the grouting effectiveness in the limestone aquifer of Qiuji Coal Mine.

## Discussion

This paper introduces a grouting effect evaluation model based on machine learning algorithms within the framework of engineering practice. This model aims to offer valuable guidance for assessing the effectiveness of grouting.

Following actual engineering comparisons, it is evident that the model’s evaluation results exhibit higher accuracy compared to traditional grouting effect assessment methods. Moreover, it considers aspects of rapidity and operability in evaluation. Furthermore, with ongoing enhancements to the dataset and algorithm optimization, its accuracy is expected to progressively improve. It is noteworthy that the method demonstrates excellent scalability and can seamlessly integrate with new data and contexts without necessitating a complete system redesign. Nonetheless, its limitations are apparent. Being a machine learning algorithm, it heavily relies on data, and issues such as insufficient data, poor data quality, or data imbalance may diminish the model's accuracy and utility. Additionally, the machine learning algorithm lacks a certain level of interpretability, posing potential challenges in certain engineering domains.

Nevertheless, this approach holds significant potential for expansion. Firstly, as researchers increasingly focus on engineering data, previously challenging-to-quantify data are being progressively delineated, paving the way for the creation of extensive “big data sets” for evaluation purposes. Secondly, various machine learning algorithms exhibit a high level of adaptability, capable of handling diverse data types such as numerical, graphical, among others. This versatility substantially enhances the practicality of machine learning algorithms. In the future, it is anticipated that grouting effect evaluation models with enhanced performance and expedited evaluation processes will be developed.

## Conclusion

1. The analysis indicates that the factors influencing the evaluation of grouting effectiveness are complex. Therefore, a system comprising “Dual-process, multi-parameter, and multi-factor” has been established.
2. The parameters of the grouting project were gathered, and an optimization-seeking algorithm and a hybrid machine learning model were developed. Following comparative analysis, it was determined that the GA-Adaboost model exhibited the best performance.
3. Engineering practice has demonstrated that the evaluation results obtained from the GA-Adaboost model are closer to the actual scenario compared to those derived from the traditional grouting effect evaluation method. This finding offers valuable insights for the development of grouting effect evaluation methodologies.

### Supplementary Information

Supplementary Information.
